# Sodium–glucose cotransporter 2 inhibitors reduce myocardial infarct size in preclinical animal models of myocardial ischaemia–reperfusion injury: a meta-analysis

**DOI:** 10.1007/s00125-020-05359-2

**Published:** 2021-01-23

**Authors:** Alex Ali Sayour, Csilla Celeng, Attila Oláh, Mihály Ruppert, Béla Merkely, Tamás Radovits

**Affiliations:** 1 Heart and Vascular Center, Semmelweis University, Budapest, Hungary; 2 University Medical Center Utrecht, Utrecht, the Netherlands

**Keywords:** Cardioprotection, Infarct size, Ischaemia–reperfusion injury, Meta-analysis, Sodium–glucose cotransporter 2 inhibitor, Systematic review

## Abstract

**Aims/hypothesis:**

Large cardiovascular outcome trials demonstrated that the cardioprotective effects of sodium–glucose cotransporter 2 (SGLT2) inhibitors might reach beyond glucose-lowering action. In this meta-analysis, we sought to evaluate the potential infarct size-modulating effect of SGLT2 inhibitors in preclinical studies.

**Methods:**

In this preregistered meta-analysis (PROSPERO: CRD42020189124), we included placebo-controlled, interventional studies of small and large animal models of myocardial ischaemia–reperfusion injury, testing the effect of SGLT2 inhibitor treatment on myocardial infarct size (percentage of area at risk or total area). Standardised mean differences (SMDs) were calculated and pooled using random-effects method. We evaluated heterogeneity by computing *Τ*^2^ and *I*^2^ values. Meta-regression was performed to explore prespecified subgroup differences according to experimental protocols and their contribution to heterogeneity was assessed (pseudo-*R*^2^ values).

**Results:**

We identified ten eligible publications, reporting 16 independent controlled comparisons on a total of 224 animals. Treatment with SGLT2 inhibitor significantly reduced myocardial infarct size compared with placebo (SMD = −1.30 [95% CI −1.79, −0.81], *p* < 0.00001), referring to a 33% [95% CI 20%, 47%] difference. Heterogeneity was moderate (*Τ*^2^ = 0.58, *I*^2^ = 60%). SGLT2 inhibitors were only effective when administered to the intact organ system, but not to isolated hearts (*p* interaction <0.001, adjusted pseudo-*R*^2^ = 47%). While acute administration significantly reduced infarct size, chronic treatment was superior (*p* interaction <0.001, adjusted pseudo-*R*^2^ = 85%). The medications significantly reduced infarct size in both diabetic and non-diabetic animals, favouring the former (*p* interaction = 0.030, adjusted pseudo-*R*^2^ = 12%). Treatment was equally effective in rats and mice, as well as in a porcine model. Individual study quality scores were not related to effect estimates (*p* = 0.33). The overall effect estimate remained large even after adjusting for severe forms of publication bias.

**Conclusions/interpretation:**

The glucose-lowering SGLT2 inhibitors reduce myocardial infarct size in animal models independent of diabetes. Future in vivo studies should focus on clinical translation by exploring whether SGLT2 inhibitors limit infarct size in animals with relevant comorbidities, on top of loading doses of antiplatelet agents. Mechanistic studies should elucidate the potential relationship between the infarct size-lowering effect of SGLT2 inhibitors and the intact organ system.

**Graphical abstract:**

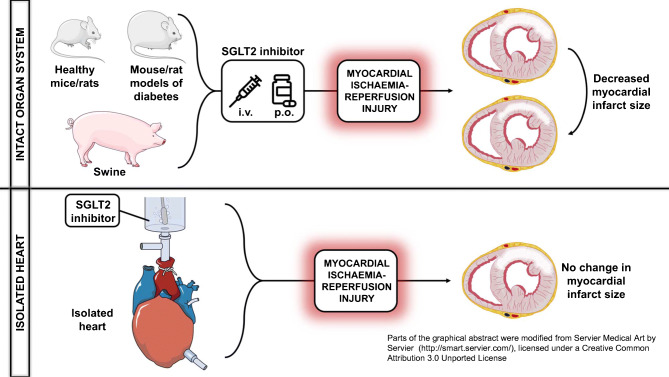

**Supplementary Information:**

The online version contains peer-reviewed but unedited supplementary material available at 10.1007/s00125-020-05359-2.

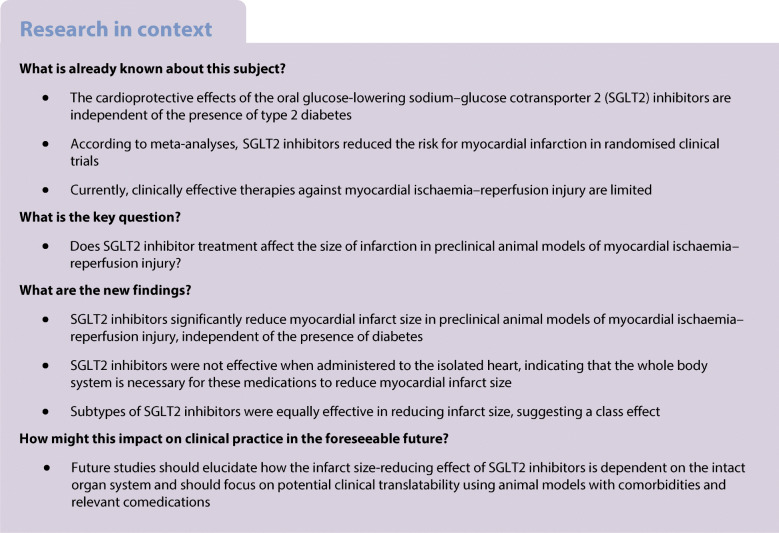

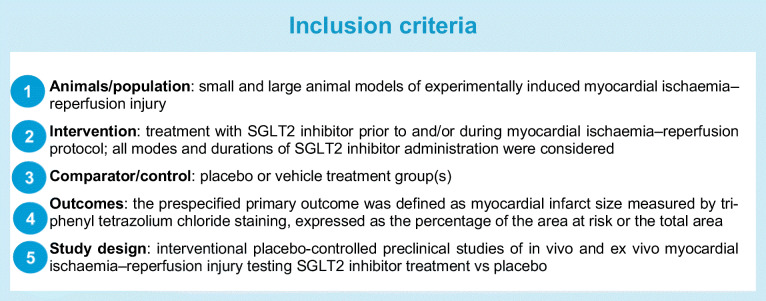

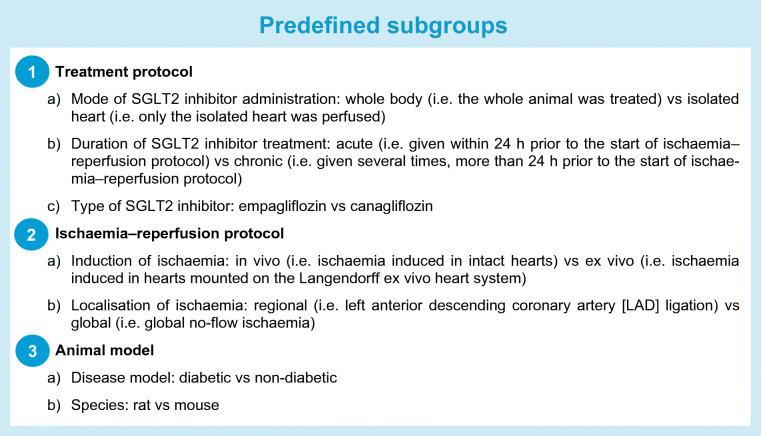







## Introduction

Sodium–glucose cotransporter 2 (SGLT2) inhibitors are novel oral glucose-lowering agents originally designed for patients with type 2 diabetes mellitus to improve glycaemic control. Their glucose-lowering action is based on the blockade of SGLT2 in the first segment of the proximal convoluted tubule in the kidney, resulting in glucosuria [1]. To date, four large cardiovascular outcome trials have reported that SGLT2 inhibitors were superior to placebo in individuals with type 2 diabetes [2–5]. Based on these trials, a meta-analysis comprising 38,723 individuals showed that SGLT2 inhibitor treatment in patients with type 2 diabetes was associated with an overall 32% reduction in hospitalisation for heart failure, whereas cardiovascular death was decreased by 17% and all-cause mortality by 15% [6]. Furthermore, SGLT2 inhibitors significantly reduced the risk for myocardial infarction by 12% [6]. Importantly, dedicated heart failure trials [7–9] demonstrated the efficacy of SGLT2 inhibitors in individuals who had heart failure with reduced ejection fraction (HFrEF), with and without type 2 diabetes, suggesting that the salutary effects of these agents are not confined to diabetic conditions.

Ischaemic heart disease is the leading cause of death worldwide, and frequently manifests in myocardial infarction. Timely reperfusion is the single most effective intervention to limit ischaemic injury [10]. However, reperfusion itself can independently induce cardiomyocyte death, increasing initial infarct size, a phenomenon termed reperfusion injury [10]. Currently, effective treatments against reperfusion injury are limited, urging the need for the development of novel therapies, since myocardial infarct size is strongly associated with mortality [11]. Due to their high efficacy, excellent tolerability and their ability to reduce major adverse cardiovascular events in large clinical trials, SGLT2 inhibitors have been tested in a variety of preclinical studies and were shown to reduce acute myocardial ischaemia–reperfusion injury in most cases [12]. In non-diabetic and diabetic animals with acute ischaemia–reperfusion injury, SGLT2 inhibitor treatment preserved left ventricular function, activated cardioprotective signalling pathways, exerted antioxidative and anti-inflammatory effects, and ameliorated mitochondrial dysfunction [13–20]. This last effect was also documented in studies which showed no infarct size-limiting effect [21, 22]. Because SGLT2 expression is negligible in the heart under normal and pathological conditions [23, 24], off-target mechanisms have been suggested [1, 12, 25, 26].

However, several other candidate drugs that were promising in preclinical models of myocardial infarction failed to impress in randomised controlled trials in humans [27]. This underscores the need for standardised, reproducible, high-quality preclinical studies that test candidate drugs in clinically relevant setups [27], as well as rigorous preclinical meta-analyses that evaluate the efficacy or inefficacy of treatments in an unbiased manner [28]. Accordingly, we aimed to review and analyse preclinical studies that tested the efficacy of SGLT2 inhibitor treatment against myocardial infarct size compared with placebo, in small and large animal models of myocardial ischaemia–reperfusion injury.

## Methods

The present systematic review and meta-analysis was carried out and interpreted in line with the Preferred Reporting Items for Systematic Reviews and Meta-Analyses (PRISMA) guideline [29]. The methodology was prespecified and published in the International Prospective Register of Systematic Reviews – (PROSPERO) (CRD42020189124). The review question was: Does SGLT2 inhibitor treatment affect the size of infarction in preclinical animal models of myocardial ischaemia–reperfusion injury?

### Search strategy

PubMed, Web of Science and Google Scholar were comprehensively searched by two independent researchers (AAS and AO) from inception to 16 June 2020 (see full search strategy in electronic supplementary material [ESM]: Search strategy and data extraction). A manual reference check of included articles was performed to identify additional articles missed by our systematic search. Only English articles published in peer-reviewed journals were considered without date restriction.

### Study selection

After the removal of duplicates, the title, abstract and full text of articles were screened to identify those fulfilling the inclusion criteria, as prespecified in the published protocol, in line with the PICOS [29] approach (see Text box ‘Inclusion criteria’). Subgroups were predefined as described in the Text box ‘Predefined subgroups’.

Exclusion criteria were as follows:

1. In vitro or invertebrate animal models of myocardial ischaemia–reperfusion injury, or experimental heart transplantation as a model of global ischaemia–reperfusion injury.
2. Administration of agents (other than anaesthetics or anticoagulants) in addition to SGLT2 inhibitors that are well-documented to alter myocardial infarct size or drug effect.
3. Absence of placebo or vehicle treatment group(s).
4. Infarct size measured in organ other than the heart, or measured with methods other than triphenyl tetrazolium chloride staining, or when not expressed as percentage of the area at risk or total area.

### Data extraction

Data extraction was performed by AAS and MR checked the integrity of the extracted data (ESM Table 1). No calibration exercise was performed, since we used a slightly modified data extraction sheet as previously described [28]. Screening for eligibility was conducted in an unblinded, standardised manner. Disagreements between the collaborators were resolved by consensus, or by arbitration from the senior author.

For the primary outcome (myocardial infarct size), we identified all individual comparisons in which a group of animals receiving an SGLT2 inhibitor was compared with a placebo/vehicle treatment group. When the primary outcome was presented graphically only, we contacted the corresponding author of the given article with a request to provide the data numerically.

### Study quality and within-study bias

Two reviewers (AAS and AO) independently assessed the study quality of each included study. Any disagreement was resolved by involving a third author (CC). We used a slightly modified Collaborative Approach to Meta-Analysis and Review of Animal Data from Experimental Studies (CAMARADES) check list (validated for quality assessment of preclinical studies [30]) to quantify the reporting of key study quality indicators, according to the prespecified adaptations as follows: ‘blinded induction of ischaemia’ was changed to ‘statement of confirmation of ischaemia’; ‘use of anaesthetic without significant intrinsic neuroprotective activity’ was changed to ‘measurement of cardiac function during ischaemia–reperfusion protocol’. Study quality score was entered into the meta-regression model as covariate to assess whether the quality of the studies influenced the effect sizes. We assumed that in ex vivo global ischaemia–reperfusion injury models, confirmation of ischaemia was self-evident.

### Data synthesis

A meta-analysis was performed for outcomes reported by at least three independent comparisons. Because of the expected high variance in infarct sizes of groups across studies, we prespecified to use standardised mean differences (SMDs), more precisely standardised difference in means, rather than weighted mean differences (WMDs) as the effect measure of the primary outcome (myocardial infarct size expressed as percentage of the area at risk or total area). Effect size (Cohen’s *d*) was calculated according to the individual comparisons between the placebo and the SGLT2 inhibitor treatment groups. The effect measure of Cohen’s *d* was corrected for small sample bias, yielding Hedges’ *g* (i.e. SMD), and 95% CIs were calculated.

In a subset of one study (Uthman et al 2019, no. 2) [22], two different doses of the SGLT2 inhibitor empagliflozin were tested on two separate groups but only one group served as a placebo control. In this case, we statistically combined the two treatment groups to create a single pairwise comparison (placebo group vs combined treatment group) as suggested by the Cochrane Handbook [31].

In another study [15], one control group was compared with three groups, in which SGLT2 inhibitor treatment differed in the time point of administration (given pre-ischaemia, during ischaemia, or at reperfusion). We combined these three treatment groups since only one group served as control.

Data were pooled using a random-effects model (Hedges’ method), due to expected between-study heterogeneity [32]. We assessed heterogeneity by calculating *I*^2^ and *Τ*^2^. We considered the following: *I*^2^ = 0–25% very low; *I*^2^ = 25–50% low; *I*^2^ = 50–75% moderate; *I*^2^>75% high heterogeneity.

We sought to explore the potential sources of heterogeneity by performing univariate meta-regression, according to the predefined subgroups (see above). These predefined variables were entered into the model separately, interaction *p* values were calculated, and the residual heterogeneity (*I*^2^ and *Τ*^2^) was assessed. To quantify the contribution of each grouping variable to heterogeneity, we calculated adjusted pseudo-*R*^2^ values.

Prespecified analyses were performed via R 3.6.3 (R Foundation for Statistical Computing, Vienna, Austria; http://www.R-project.org/) using the ‘metafor’ package [33] on JASP 0.12.2 (JASP Team, https://jasp-stats.org) and via SPSS 25 (IBM, Armonk, NY, USA) with syntax from Field and Gillett [34]. GraphPad Prism 8 (GraphPad Software, San Diego, CA, USA) was used to depict data. A *p* value of <0.05 was considered statistically significant.

### Risk of bias across studies

We constructed a funnel plot to explore the relationship between effect estimates (SMDs) and the measure of precision (SE of SMDs). Funnel plot asymmetry was assessed according to Egger’s regression test as well as using a modified ranked correlation test (based on Kendall’s *T*).

Funnel plot asymmetry can result from a number of issues including, but not limited to, publication bias. Because we tested a hypothesis that a drug therapy might favourably affect an outcome, we considered the presence of one-tailed publication bias (i.e. pattern of selection that favours the publication of studies reporting significant positive effects). To correct the population effect estimate for moderate and severe publication bias, a priori weight functions as per Vevea and Woods [34, 35] were applied. Second, we performed a trim-and-fill analysis which identifies ‘missing studies’ and accordingly adjusts the population effect estimate for publication bias, which is therefore considered bias-corrected [35].

### Sensitivity analysis

We performed a prespecified sensitivity analysis to ensure the robustness of our calculations. First, we pooled estimates of WMDs of the primary outcome to explore whether this yielded results comparable with SMDs. Second, we pooled the SMDs using the fixed-effects model to explore whether it produced an overall estimate that corresponded to that derived from the random-effects model.

## Results

### Study selection and characteristics

**Fig. 1:**
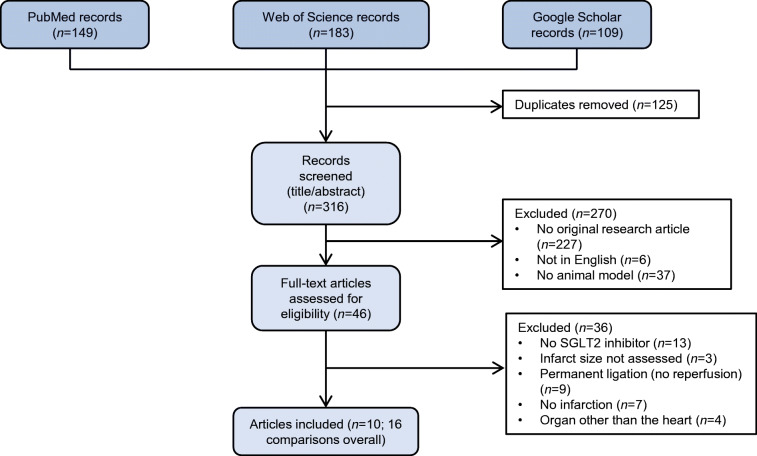
Flow chart of the study identification and selection process. A systematic review yielded 316 unique records as of 16 June 2020. After application of inclusion and exclusion criteria, a total of ten eligible studies were identified reporting 16 individual comparisons, which were included in the meta-analysis

The predefined comprehensive search strategy identified 316 unique records, of which 270 were excluded based on title and abstract (Fig. 1). After assessing 46 full-text articles, 36 were excluded due to inclusion criteria not being met. In total, ten articles [13–22] reporting an overall of 16 independent controlled comparisons on SGLT2 inhibitor treatment vs placebo in myocardial ischaemia–reperfusion injury met the prespecified inclusion criteria (Fig. 1). We analysed data from 101 control animals and 123 animals treated with SGLT2 inhibitors.

We contacted six authors, five of whom responded and supplied the missing numerical data. For the remaining study, we digitally scanned the relevant graph, calibrated the axes and extracted the data.

All included studies and their extracted data are presented in ESM Table 1.

### Study quality

**Fig. 2:**
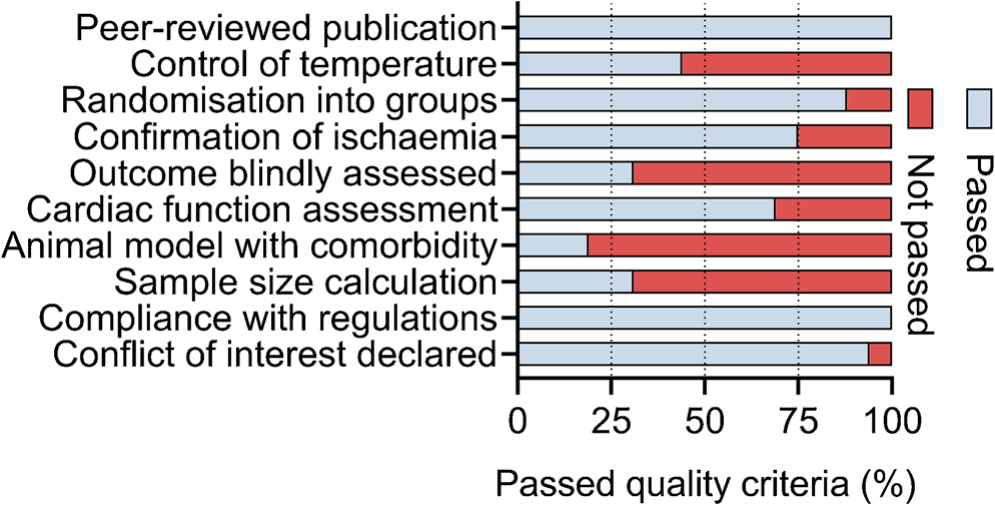
Exploration of within-study bias. The prespecified modified form of the CAMARADES validated checklist was used to assess study quality. The list consists of the depicted ten points, which were evaluated for each individual comparison

The median study quality score of the included studies was 6.5 (IQR 6–7) out of 10 (Fig. 2), indicating that some study quality indicators were poorly reported (Fig. 2). Individual study quality scores are shown in ESM Table 2.

None of the involved studies reported adverse events related to SGLT2 inhibitor treatment.

### Meta-analysis

#### Primary outcome and heterogeneity

**Fig. 3:**
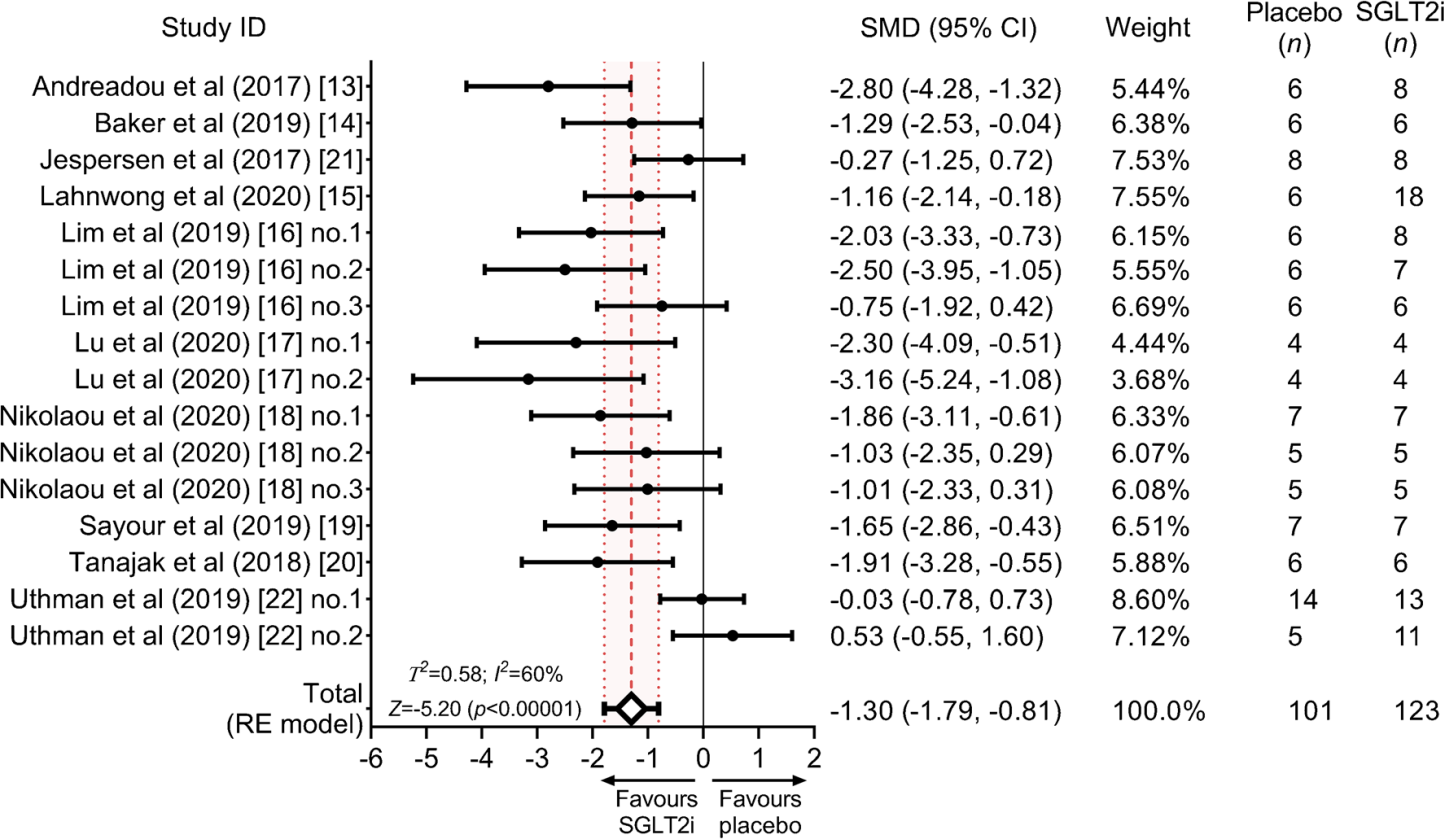
Forest plot of the size of effect of SGLT2 inhibitor treatment on myocardial infarct size vs placebo. Myocardial infarct size (% area at risk or total area) is quantified as SMD (black circles) and 95% CIs. The pooled effect estimate is shown as a diamond and 95% CIs are depicted. The dashed line represents the total pooled estimate and the shading, bounded by dotted lines, depicts its 95% CI. RE, random-effects; SGLT2i, SGLT2 inhibitor

Overall, SGLT2 inhibitor treatment, as compared with placebo, significantly reduced myocardial infarct size (SMD = −1.30 [95% CI −1.79, −0.81], *Z* = −5.20, *p* < 0.00001) (Fig. 3), referring to a mean 33% (95% CI 20%, 47%) difference in ratios. We observed moderate heterogeneity (*I*^2^ = 60%, *Τ*^2^ = 0.58), which was significant (*Q* = 39.46, *p* < 0.001) (Fig. 3).

#### Subgroup analysis

**Fig. 4:**
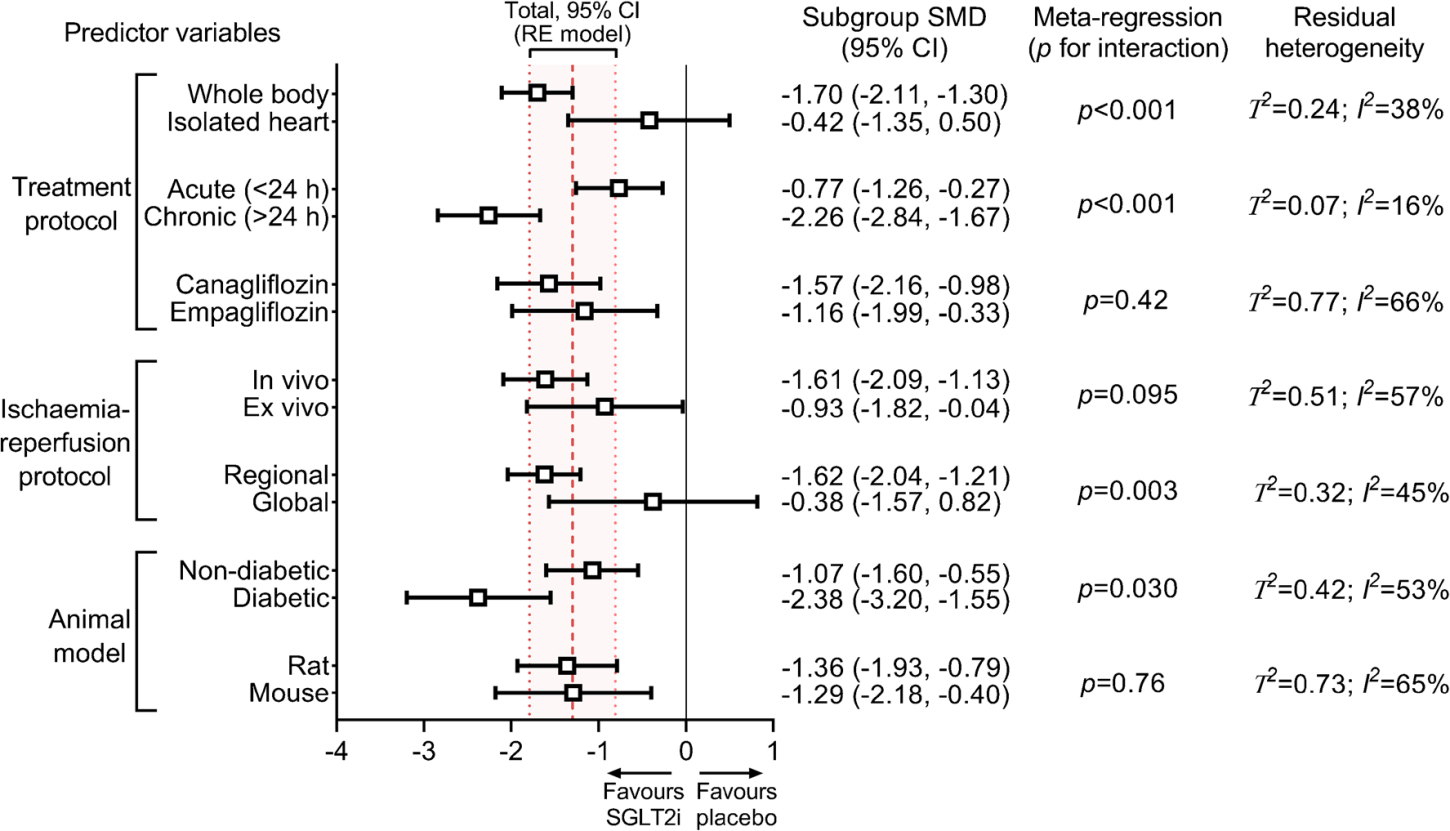
Impact of experimental factors on the infarct size-lowering effect of SGLT2 inhibitors. SMDs according to prespecified subgroups are depicted. Significance of interactions is shown according to univariate meta-regression, without correction for multiple comparisons. Residual heterogeneities are reported according to each experimental variable. The dashed line represents the total pooled estimate and the shading, bounded by dotted lines, depicts its 95% CI, corresponding to data shown in the forest plot (Fig. 3). In line with the prespecified protocol, we only performed meta-analysis on at least three independent comparisons. Hence, dapagliflozin could not be included in the comparison regarding the efficacy of SGLT2 inhibitor subtypes. Furthermore, only one study tested SGLT2 inhibitor in swine, therefore it is excluded from the species subgroups. RE, random-effects

Significant infarct size reduction was observed only when SGLT2 inhibitors were administered to the intact organ system and not to isolated hearts (*p* for interaction <0.001, adjusted pseudo-*R*^2^ = 47%) (Fig. 4). Both acute and chronic administration of SGLT2 inhibitors resulted in significant infarct size reduction but with significant between-group difference (*p* for interaction <0.001, adjusted pseudo-*R*^2^ = 85%) (Fig. 4). Empagliflozin and canagliflozin similarly reduced myocardial infarct size (*p* for interaction = 0.42) (Fig. 4); only two studies assessed dapagliflozin.

Regarding the ischaemia–reperfusion protocol, in vivo vs ex vivo induction of ischaemia did not result in significant differences in total effect size estimates (*p* for interaction = 0.095) (Fig. 4). SGLT2 inhibitors potently reduced infarct size in regional ischaemia–reperfusion models but not in global models (*p* for interaction = 0.003, adjusted pseudo-*R*^2^ = 33%) (Fig. 4). Additional post hoc analyses related to the ischaemia–reperfusion protocol are provided in ESM Table 3.

Compared with placebo, SGLT2 inhibitor treatment significantly reduced infarct size in both diabetic and non-diabetic animals (Fig. 4). However, the effect was moderately larger in diabetic animals (*p* for interaction = 0.030, adjusted pseudo-*R*^2^ = 12%) (Fig. 4). No subgroup differences were observed among the different species (rat vs mouse, *p* for interaction = 0.76) (Fig. 4); only one study thus far used a porcine model.

We found no association between study quality scores and effect estimates (*p* = 0.33).

### Risk of bias across studies

**Fig. 5:**
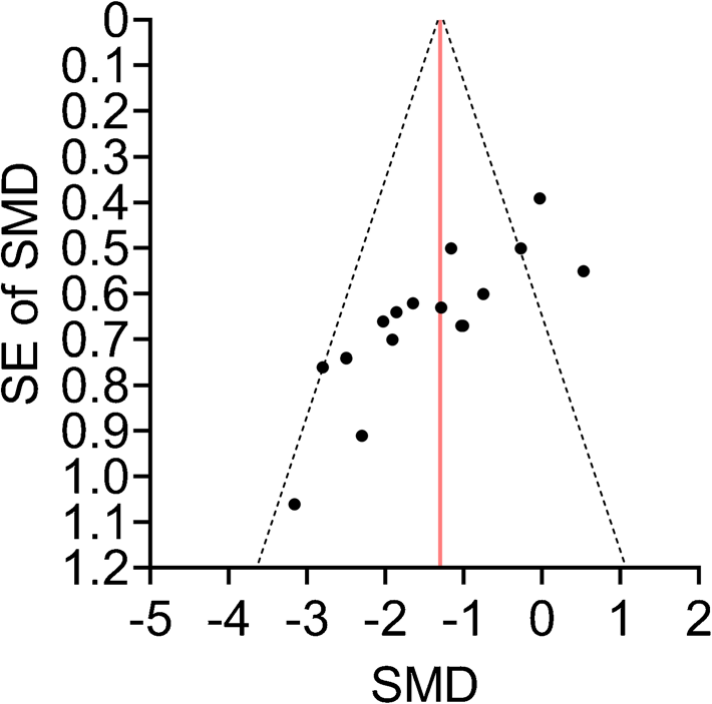
Funnel plot depicting SMDs plotted against their measure of precision (SE). The vertical line represents the total pooled estimate corresponding to that on the forest plot (Fig. 3). Its pseudo-95% CIs are depicted with dashed lines. Note that two points appear to be optically fused because of similar SMDs (−1.01 and −1.03) and the same SE (0.67)

**Fig. 6:**
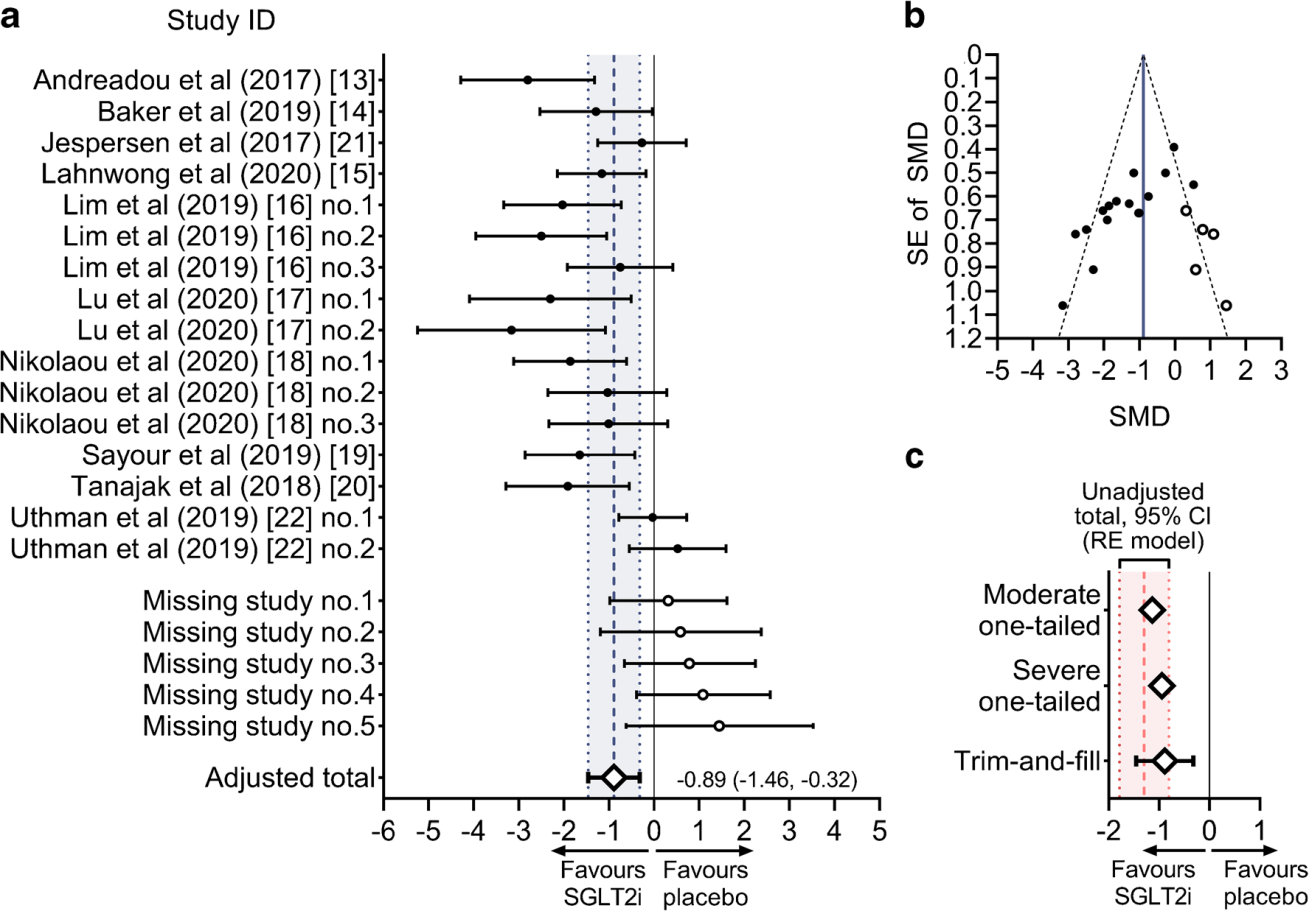
Exploration and adjustment for publication bias. (**a**, **b**) Trim-and-fill analysis showing the modified forest plot and funnel plot with values according to the theoretically missing studies (process called ‘filling’; white circles). Note that on the funnel plot, two points appear to be optically fused because of similar SMDs (−1.01 and −1.03) and a same SE (0.67). (**c**) Summary of methods used to explore and adjust for publication bias. A priori weight functions were applied to simulate moderate and severe one-tailed selection (i.e. pattern of selection that tends to favour the publication of studies reporting significant positive effects). The adjusted summary estimate from the trim-and-fill analysis is also depicted. The unadjusted summary estimate is shown as a dashed line and the shading, bounded by dotted lines, depicts its 95% CI, corresponding to data shown in the forest plot (Fig. 3). RE, random-effects

Visual inspection of funnel plot revealed asymmetry (Fig. 5). Both Egger’s regression test (*Z* = −5.06, *p* < 0.001) and the rank correlation test (Kendall’s *Τ* = −0.633, *p* < 0.001) signalled the presence of small-study effect. We estimated that the adjusted overall effect was SMD = −1.14 in case of moderate, and SMD = −0.95 in case of severe one-tailed selection (Fig. 6c). Both values corresponded to our unadjusted total effect and its 95% CIs (Fig. 6c).

The trim-and-fill analysis identified that, theoretically, five studies were missing from the analysis (Fig. 6a, b). Accordingly, the estimated bias-corrected overall effect was large and significant (SMD = −0.89 [95% CI −1.46, −0.32], *p* = 0.002), corresponding to our overall unadjusted effect estimate (Fig. 6a, b).

### Sensitivity analysis

When pooling the estimates of the primary outcome as unstandardised WMDs rather than SMDs, we found a similarly significant reduction in infarct size associated with SGLT2 inhibitor treatment compared with placebo (WMD = −10.90% [95% CI −15.03%, −6.78%], *Z* = −5.18, *p* < 0.00001) (ESM Fig. 1), with high heterogeneity (*Τ*^2^ = 50.30, *I*^2^ = 80%). When pooling SMDs using a fixed-effects model instead of a random-effects model, the overall effect estimate was still large and significant (SMD_fixed_ = −1.09 [95% CI −1.38, −0.79], *Z* = −7.10, *p* < 0.00001), with substantially narrower 95% CIs, as expected (ESM Fig. 2). Heterogeneity was moderate (*I*^2^ = 62%) and comparable with that estimated using the random-effects model.

## Discussion

In this meta-analysis of preclinical studies, we found that the glucose-lowering SGLT2 inhibitors reduce myocardial infarct size, independently of the presence of type 2 diabetes. This effect depends on the mode of administration as SGLT2 inhibitors seem to be effective only when given to the intact organ system, and not to isolated hearts.

Ischaemic heart disease is the leading cause of death worldwide, and myocardial infarction is its major manifestation [10]. Myocardial infarct size is a strong and independent predictor of all-cause mortality, hospitalisation for heart failure, and reinfarction within 1 year [11]. Therefore, reducing the size of infarct is highly relevant in improving outcomes and life expectancy [36]. While several candidate drugs and treatment approaches have been promising in preclinical studies, the bench-to-bedside approach has mostly been disappointing [27, 37]. Given their excellent cardiovascular safety and efficacy in randomised clinical trials, SGLT2 inhibitors have been the subject of a growing number of preclinical studies investigating their effect on myocardial infarct size, representing an emerging ‘bedside-to-bench’ approach [12].

In this meta-analysis of preclinical studies, we show that SGLT2 inhibitors significantly reduce myocardial infarct size. This refers to an overall difference of 33% in infarct size/area at risk or total area ratios. We observed, however, subgroup differences that might be explained by distinct study designs. Most importantly, we found that SGLT2 inhibitors did not significantly reduce myocardial infarct size when administered to isolated hearts, whereas they were highly effective when administered to the intact organ system. This is in line with the mechanistic review of Andreadou et al [12], who speculated that the infarct size-mitigating effect of SGLT2 inhibitors is dependent on the whole body system.

There are several crucial differences between isolated and intact hearts in terms of ischaemia–reperfusion injury, possibly explaining the above finding. First, Langendorff ex vivo hearts are not subject to physiological loading conditions [27]. SGLT2 inhibitors have direct vasodilatory effects [38] and moderately reduce afterload [19], which could contribute to infarct size-lowering in intact hearts. Second, ex vivo hearts are perfused with solutions devoid of ketones. However, while SGLT2 inhibitors increase circulating levels of ketone bodies in individuals with diabetes [39], they have no such effect in non-diabetic conditions and do not increase myocardial ketone uptake [14]. Third, ex vivo hearts completely lack innervation and are not subject to circulating hormonal effectors. Nevertheless, SGLT2 inhibitors seem to have a mode of action other than that of renin–angiotensin–aldosterone system inhibitors, given their additivity [8, 9]. Fourth, kidneys are absent in ex vivo settings, therefore it is unclear whether renal effects contribute to cardioprotection, especially given the fact that renoprotection and cardioprotection are closely intertwined in the case of SGLT2 inhibitors [5]. Finally, arguably the most clinically important difference is that isolated hearts lack circulating immune cells and thrombocytes, which play key roles in myocardial ischaemia–reperfusion injury [40–42]. SGLT2 inhibitors have potential anti-inflammatory effects [43], as noted in studies that showed infarct size reduction [17, 18]. Furthermore, SGLT2 inhibitors exert an antiplatelet action via blocking ADP-stimulated platelet activation [44]. ADP receptor antagonists (e.g. clopidogrel) that are routinely administered to patients with acute myocardial infarction also have direct infarct size-reducing effects, independent of antiplatelet function [45]. Hence, Cohen and Downey postulated that only those medications that affect myocardial infarct size through mechanistic pathways other than those modulated by antiplatelet agents could be meaningfully cardioprotective in the clinical setting [42]. Unfortunately, none of the included studies in the present meta-analysis reported the use of antiplatelet therapy as co-medication, so whether SGLT2 inhibitors reduce myocardial infarct size additionally to ADP receptor antagonists still needs to be elucidated.

Based on the above, SGLT2 inhibitors might reduce myocardial infarct size through indirect effects. In the studies of Lim (no. 1 and no. 2) [16], rats were chronically treated with SGLT2 inhibitor, then hearts were isolated and the induction of ischaemia was performed ex vivo. The SGLT2 inhibitor-treated hearts (independent of diabetes) showed considerably smaller infarct sizes compared with placebo-treated hearts [16]. Therefore, it seems that the whole body system is required for SGLT2 inhibitors to reduce infarct size but that the cardioprotective signal is retained in the heart independent of diabetes. Interestingly, SGLT2 inhibitors induced molecular changes in healthy (non-ischaemic) hearts similar to those in treated hearts with ischaemia–reperfusion injury [18, 19]. This might suggest a potential conditioning phenomenon. Chronic administration of SGLT2 inhibitors could be more effective in evoking these beneficial cardiac signals, possibly accounting for the greater infarct size reduction as compared with acute treatment. In clinical trials, diabetic patients receiving long-term SGLT2 inhibitor treatment had a higher chance of surviving myocardial infarction than those receiving placebo [46]. Therefore, whether these agents exert similar cardioprotection in high-risk non-diabetic individuals warrants further elucidation.

We found that SGLT2 inhibitor treatment significantly reduced infarct size independent of whether ischaemia was induced in vivo or ex vivo. While SGLT2 inhibitors effectively mitigated infarct size only in studies with regional ischaemia (LAD ligation), Andreadou et al [12] postulated that protocols with global no-flow ischaemia showed neutral results because SGLT2 inhibitors could only be administered ex vivo. Taking all these findings into consideration, head-to-head comparisons are needed to identify the presence of factors in the intact organ system that facilitate the infarct size-reducing effect of SGLT2 inhibitors, which are absent from isolated heart settings. These findings could potentially contribute to our understanding of the mechanisms underlying the diverse cardioprotective actions of these agents.

We showed that the glucose-lowering SGLT2 inhibitors were equally effective in mitigating myocardial infarct size in diabetic and non-diabetic animals, though the effect was larger when diabetes was present. Interestingly, SGLT2 inhibitors activated similar cardioprotective mediators in diabetic animals as in non-diabetic animals [13, 15, 18, 20]. This might further confirm that SGLT2 inhibitors have cardioprotective effects that are at least in part independent of the amelioration of the deranged diabetic milieu [1, 46, 47], in line with recent dedicated heart failure trials [7–9]. However, ketamine and xylazine, widely used in preclinical studies, severely increase blood glucose levels in small animals [48], which might increase the size of infarction per se [49]. Given that SGLT2 inhibitors can normalise acute hyperglycaemia, future studies should carefully select the anaesthetic regimen when investigating cardioprotection and should report blood and urinary glucose levels. Nevertheless, studies in non-diabetic rats [16, 19], mice [17, 18] and swine [14] reported that SGLT2 inhibitor treatment (either acute or chronic) did not affect blood glucose levels. The medications were equally effective in these species, with the only study thus far using a porcine model reporting significant infarct size reduction. The latter is promising in terms of clinical translatability [27].

There was no difference in the infarct size-mitigating capability of canagliflozin and empagliflozin; only two studies have tested dapagliflozin so far, both reporting significantly positive results. In cardiovascular outcome trials, SGLT2 inhibitors were consistently effective, especially in terms of reducing the number of hospitalisations for heart failure [6, 50], indicating a class effect. Interestingly, the selectivity of SGLT2 inhibitors for SGLT2 over SGLT1 shows high variation, with canagliflozin being the least selective. It is unlikely that SGLT2 inhibitors, in clinically achievable plasma concentrations, would block SGLT1 in the heart [51], although SGLT1 is upregulated in myocardial ischaemia [52]. Notably, SGLT1 knockdown protects against in vivo myocardial [52] and renal [53] ischaemia–reperfusion injury. Future studies testing the dual SGLT1/2 inhibitor sotagliflozin in acute myocardial ischaemia–reperfusion injury are warranted and could reveal the differences in mechanism of action as compared with SGLT2 inhibitors.

We quantified within-study bias by using a score system validated for small animal studies. More than half of the included studies reported assessment of cardiac function. Beside the overall lack of sample size calculations (hence, statistical power [27]), less than half of the studies reported that temperature was controlled. The infarct size-modulating effect of hypo- and hyperthermia is well-characterised [54], and this could distort the effect of a medication. Finally, fewer than one-third of the studies reported that infarct size was assessed blindly, presenting a considerable limitation [27]. Nonetheless, study quality scores were not related to effect size estimates, suggesting that study quality did not explain the effect of SGLT2 inhibitors on infarct size.

We assessed between-study bias by constructing a funnel plot, which suggested that small, neutral studies were under-represented in our analysis. However, funnel plot asymmetry needs to be cautiously interpreted when the number of studies involved is relatively low. Also, asymmetry can arise from a number of issues including but not limited to publication bias [35]. Because the included studies tested the effect of a medication on a primary outcome, we could not rule out the occurrence of significance-based publication bias (i.e. selective reporting of significantly positive studies only). To analyse this issue, we applied a priori weight functions according to moderate and severe one-tailed selections [34, 35], and performed trim-and-fill analysis, all showing that the overall effect estimate would not be substantially altered in case of publication bias that favoured the publication of significantly positive results only.

### Limitations

Several aspects limit our present meta-analysis, as its validity is subject to the quality of the reporting of the included studies. Only articles in English could be included. One limitation is the moderate number of included studies; nonetheless, data are reported on a relatively large number of animals (more than 200). Another limitation is the moderate heterogeneity; however, this is expected in preclinical studies [32]. Furthermore, although we cannot exclude the presence of publication bias, we provide three estimates that adjust for it. For two of the comparisons (Lahnwong et al and Uthman et al no. 2), treatment groups were combined because only one group served as control; this might be suboptimal but it is the recommended approach [31].

None of the studies investigated the effect of SGLT2 inhibitors alongside relevant comorbidities or comedications that are routinely administered in individuals with myocardial infarction, and all studies used only young animals. Three studies contained animals with diabetes, although these models differed vastly in mode of induction. Only male animals were included, therefore whether cardioprotection could be achieved in female counterparts needs to be elucidated. Nonetheless, SGLT2 inhibitors are equally cardioprotective in male and female humans [55]. Finally, only one large animal study tested the effect of SGLT2 inhibitor treatment on myocardial infarct size thus far, reporting a positive effect.

## Conclusions and future perspectives

The glucose-lowering SGLT2 inhibitors significantly reduce myocardial infarct size in preclinical animal models of ischaemia–reperfusion injury, independent of the presence of diabetes. In the future, clinically relevant small and large animal studies with different comorbidities are needed to test whether SGLT2 inhibitors exert additional cardioprotection on top of loading doses of ADP receptor antagonist. Furthermore, for better understanding of the complex salutary effects of these agents, mechanistic studies should explore whether there is a causal relationship between the infarct size-reducing effect of SGLT2 inhibitors and the intact organ system.

## Supplementary Information

ESM 1 (PDF 271 kb)

## Data Availability

The raw datasets used during the current study are included in ESM Tables 1–3.

## Abbreviations

CAMARADES: Collaborative Approach to Meta-Analysis and Review of Animal Data from Experimental Studies
LAD: Left anterior descending coronary artery
SGLT2: Sodium–glucose cotransporter 2
SMD: Standardised mean difference
WMD: Weighted mean difference
