# Supplementary material for: Sodium–glucose cotransporter 2 inhibitors reduce myocardial infarct size in preclinical animal models of myocardial ischaemia–reperfusion injury: a meta-analysis
Source: Diabetologia. 2021 Jan 23;64(4):737–48. doi: 10.1007/s00125-020-05359-2 (PMC7940278; doi:10.1007/s00125-020-05359-2)
Supplement: Supplementary file 1 — (PDF 271 kb) [file 125_2020_5359_MOESM1_ESM.pdf]

Electronic Supplementary Material (ESM)

Search strategy and data extraction

The search strategy was developed by AAS and TR, which was peer-reviewed by one member of the research group. PubMed, Web of Science, and Google Scholar were comprehensively searched by two independent researchers (AAS and AO) from inception to June 16, 2020. Duplicates were removed using the online version of EndNote (Clarivate, Philadelphia, PA, USA).

1. PubMed

We used the following search terms in PubMed, with incorporation of MeSH Terms and translations:

Search: ((((("sodium glucose transporter 2 inhibitors"[Pharmacological Action] OR "sodium-glucose transporter 2 inhibitors"[MeSH Terms]) OR "sodium glucose transporter 2 inhibitors"[All Fields]) OR ("sglt2"[All Fields] AND "inhibitor"[All Fields])) OR "sglt2 inhibitor"[All Fields]) AND ((((((((("infarctation"[All Fields] OR "infarcted"[All Fields]) OR "infarctic"[All Fields]) OR "infarcting"[All Fields]) OR "infarction"[MeSH Terms]) OR "infarction"[All Fields]) OR "infarct"[All Fields]) OR "infarctions"[All Fields]) OR "infarcts"[All Fields]) OR "infarctive"[All Fields]) OR ("isch*"[All Fields] AND (((((((("reperfusate"[All Fields] OR "reperfusates"[All Fields]) OR "reperfuse"[All Fields]) OR "reperfused"[All Fields]) OR "reperfusing"[All Fields]) OR "reperfusion"[MeSH Terms]) OR "reperfusion"[All Fields]) OR "reperfusions"[All Fields]) OR "reperfusive"[All Fields])))) AND ((((("heart"[MeSH Terms] OR "heart"[All Fields]) OR "hearts"[All Fields]) OR "heart s"[All Fields]) OR ((("cardiacs"[All Fields] OR "heart"[MeSH Terms]) OR "heart"[All Fields]) OR "cardiac"[All Fields])) OR ((("myocardially"[All Fields] OR "myocardium"[MeSH Terms]) OR "myocardium"[All Fields]) OR "myocardial"[All Fields])) Filters: Journal Article

2. Web of Science

We searched Web of Science as follows:

TOPIC: (SGLT2 inhibitor) *AND* TOPIC: (infarction OR isch* reperfusion) *AND* TOPIC: (heart OR cardiac OR myocardial)

Refined by: DOCUMENT TYPES: (Article)

Databases: WOS, BCI, CSCD, CCC, DRCI, DIIDW, KJD, MEDLINE, RSCI, SCIELO, ZOOREC. Search language=Auto

3. Google Scholar

We used the following search terms in Google Scholar:

"SGLT2 inhibitor" AND "ischemia reperfusion" AND "heart" AND "infarct size" Except: patents

STUDY ID	1	2	3	4	5			6				7		8				9			10		
					a	b	c	a	b	c	d	a	b	a	b	c	d	a	b	c	a	b	c
Andreadou et al (2017) [13]	M	M	C57Bl/6J	22 w	diab	W diet	14 w	empa	10 mg/kg/d	whole body (oral)	chronic (6 w)	i.p.: ketamine, xylazine, atropine	Oxygen	in vivo	regional	30 min	120 min	33.2	3.429	6	17.6	6.223	8
Baker et al (2019) [14]	S	M	domestic	50 kg	-	-	-	cana	300 mg	whole body (oral)	acute (24 h and 2 h)	i.v.: telazol, ketamine, xylazine, α-chloralose, morphine	N/A	in vivo	regional	60 min	120 min	8.8	4.409	6	3.7	2.694	6
Jespersen et al (2017) [21]*	R	M	Wistar	300-350 g	-	-	-	empa	2.14 mg/L	isolated heart (perf)	acute (pre-isch 10 min)	s.c.: midazolam, fluanisone, fentanyl citrate	-	ex vivo	global	40 min	120 min	60.77	14.790	8	56.02	18.470	8
Lahnwong et al (2020) [15]	R	M	Wistar	250-300 g	-	-	-	dapa	1 mg/kg	whole body (i.v.)	acute (pre-isch *or* isch *or* rep)	i.m.: zolazepam, tiletamine, xylazine	Room air	in vivo	regional	20 min	120 min	47.5	5.5	6	36.9	9.7	18
Lim et al (2019) [16] no.1	R	M	ZDF Lean	300-400 g	-	-	-	cana	166.7 mg/kg	whole body (oral)	chronic (4 w)	i.p.: pentobarbital	-	ex vivo	regional	35 min	120 min	55	17.146	6	27	8.485	8
Lim et al (2019) [16] no.2	R	M	ZDF	350-450 g	diab	HFD	4 w	cana	100 mg/kg	whole body (oral)	chronic (4 w)	i.p.: pentobarbital	-	ex vivo	regional	35 min	120 min	37	7.348	6	20	5.292	7
Lim et al (2019) [16] no.3	R	M	SD	290-350 g	-	-	-	cana	10 µmol/L	isolated heart (perf)	acute (perf)	i.p.: pentobarbital	-	ex vivo	regional	35 min	120 min	45	9.798	6	38	7.348	6
Lu et al (2020) [17] no.1	M	M	C57Bl/6J	12 w	-	-	-	empa	2.5 µmol/L	isolated heart (perf)	acute (perf)	isoflurane	-	ex vivo	global	40 min	30 min	25.1	9.820	4	5.04	4.300	4
Lu et al (2020) [17] no.2	M	M	C57Bl/6J	12 w	-	-	-	empa	10 mg/kg/d	whole body (oral)	chronic (3 d)	isoflurane	N/A	in vivo	regional	45 min	24 h	11.44	2.940	4	3.15	1.320	4
Nikolaou et al (2020) [18] no.1	M	M	C57Bl/6J	18 w	-	-	-	empa	10 mg/kg	whole body (oral)	chronic (6 w)	i.p.: ketamine, xylazine, atropine	N/A	in vivo	regional	30 min	120 min	45.8	8.466	7	29.5	7.937	7
Nikolaou et al (2020) [18] no.2	M	M	C57Bl/6J	12 w	-	-	-	empa	10 mg/kg	whole body (oral)	acute (24 h)	i.p.: ketamine, xylazine, atropine	N/A	in vivo	regional	30 min	120 min	46.8	6.261	5	40.9	3.801	5
Nikolaou et al (2020) [18] no.3	M	M	C57Bl/6J	12 w	-	-	-	empa	10 mg/kg	whole body (oral)	acute (4 h)	i.p.: ketamine, xylazine, atropine	N/A	in vivo	regional	30 min	120 min	45.8	5.590	5	39.3	6.037	5
Sayour et al (2019) [19]	R	M	SD	250-350 g	-	-	-	cana	3 µg/kg	whole body (i.v.)	acute (isch)	i.p.: pentobarbital	Oxygen	in vivo	regional	30 min	120 min	59.3	11.112	7	42.5	7.673	7
Tanajak et al (2018) [20]	R	M	Wistar	22-24 w	diab	HFD	16 w	dapa	1 mg/kg/d	whole body (oral)	chronic (4 w)	i.m.:zolazepam, tiletamine, xylazine	N/A	in vivo	regional	30 min	120 min	48	12.247	6	25	9.798	6
Uthman et al (2019) [22] no.1	M	M	C57Bl/6NCrl	10-14 w	-	-	-	empa	1 µmol/L	isolated heart (perf)	acute (perf)	i.p.: ketamine, dexmedetomidine	-	ex vivo	global	25 min	120 min	51.2	20.579	14	50.7	14.062	13
Uthman et al (2019) [22] no.2	M	M	C57Bl/6NCrl	10-14 w	-	-	-	empa	3 *or* 10 µmol/L	isolated heart (perf)	acute (perf)	i.p.: ketamine, dexmedetomidine	-	ex vivo	global	25 min	120 min	55	8.720	5	59.46	7.670	11

ESM Table 1. Extracted data – study characteristics.

1: species (M=mouse; S=swine; R=rat); 2: sex (M=male); 3: strain (SD=Sprague-Dawley; ZDF=zucker diabetic fatty rat); 4: age or weight at time of ischaemia-reperfusion protocol; 5: comorbidity, 5a: disease (diab=type 2 diabetes), 5b: induction (W=western; HFD=high fat diet), 5c: duration; 6: treatment protocol, 6a: type (empa=empagliflozin; cana=canagliflozin; dapa=dapagliflozin), 6b: dosage, 6c: mode of administration (perf=perfusate; i.v.=intravenous), 6d: duration (isch=ischaemia; perf=perfusate; rep=reperfusion); 7: surgical protocol, 7a: anaesthetic regimens (i.m.=intramuscular; i.p.=intraperitoneal; i.v.=intravenous; s.c.=subcutaneous), 7b: mode of ventilation (N/A=not detailed); 8: ischaemia-reperfusion protocol, 8a: induction, 8b: localization, 8c: duration of ischaemia, 8d: duration of reperfusion); 9: control group, 9a: mean myocardial infarct size (% area at risk or total area), 9b: standard deviation, 9c: number of animals; 10: treatment group, 10a: mean myocardial infarct size (% area at risk or total area), 10b: standard deviation, 10c: number of animals

[13] Andreadou I, Efentakis P, Balafas E, et al. (2017) Empagliflozin Limits Myocardial Infarction in Vivo and Cell Death in Vitro: Role of STAT3, Mitochondria, and Redox Aspects. Front Physiol 8: 1077. 10.3389/fphys.2017.01077

[14] Baker HE, Kiel AM, Luebbe ST, et al. (2019) Inhibition of sodium-glucose cotransporter-2 preserves cardiac function during regional myocardial ischemia independent of alterations in myocardial substrate utilization. Basic Res Cardiol 114(3): 25. 10.1007/s00395-019-0733-2

[15] Lahnwong S, Palee S, Apaijai N, et al. (2020) Acute dapagliflozin administration exerts cardioprotective effects in rats with cardiac ischemia/reperfusion injury. Cardiovasc Diabetol 19(1): 91. 10.1186/s12933-020-01066-9

[16] Lim VG, Bell RM, Arjun S, Kolatsi-Joannou M, Long DA, Yellon DM (2019) SGLT2 Inhibitor, Canagliflozin, Attenuates Myocardial Infarction in the Diabetic and Nondiabetic Heart. JACC Basic Transl Sci 4(1): 15-26. 10.1016/j.jacbts.2018.10.002

[17] Lu Q, Liu J, Li X, et al. (2020) Empagliflozin attenuates ischemia and reperfusion injury through LKB1/AMPK signaling pathway. Mol Cell Endocrinol 501: 110642. 10.1016/j.mce.2019.110642

[18] Nikolaou PE, Efentakis P, Qourah FA, et al. (2020) Chronic Empaglifozin treatment reduces myocardial infarct size in non-diabetic mice through STAT-3 mediated protection on microvascular endothelial cells and reduction of oxidative stress. Antioxid Redox Signal. 10.1089/ars.2019.7923

[19] Sayour AA, Korkmaz-Icoz S, Loganathan S, et al. (2019) Acute canagliflozin treatment protects against in vivo myocardial ischemia-reperfusion injury in non-diabetic male rats and enhances endothelium-dependent vasorelaxation. J Transl Med 17(1): 127. 10.1186/s12967-019-1881-8

[20] Tanajak P, Sa-Nguanmoo P, Sivasinprasasn S, et al. (2018) Cardioprotection of dapagliflozin and vildagliptin in rats with cardiac ischemia-reperfusion injury. J Endocrinol 236(2): 69-84. 10.1530/JOE-17-0457

[21] Jespersen NR, Lassen TR, Hjortbak MV, et al. (2017) Sodium Glucose Transporter 2 (SGLT2) Inhibition does not Protect the Myocardium from Acute Ischemic Reperfusion Injury but Modulates Post-Ischemic Mitochondrial Function. Cardiovascular Pharmacology: Open Access 6(2). 10.4172/2329-6607.1000210

[22] Uthman L, Nederlof R, Eerbeek O, et al. (2019) Delayed ischaemic contracture onset by empagliflozin associates with NHE1 inhibition and is dependent on insulin in isolated mouse hearts. Cardiovasc Res 115(10): 1533-1545. 10.1093/cvr/cvz004

STUDY ID	1	2	3	4	5	6	7	8	9	10	Overall score
**Andreadou et al (2017) [13]**	Y	-	Y	Y	-	-	Y	-	Y	Y	6
**Baker et al (2019) [14]**	Y	-	Y	-	Y	Y	-	-	Y	Y	6
**Jespersen et al (2017) [21]***	Y	-	-	Y	-	-	-	-	Y	-	3
**Lahnwong et al (2020) [15]**	Y	-	Y	Y	Y	Y	-	-	Y	Y	7
**Lim et al (2019) [16] no.1**	Y	Y	Y	-	-	Y	-	Y	Y	Y	7
**Lim et al (2019) [16] no.2**	Y	Y	Y	-	-	Y	Y	Y	Y	Y	8
**Lim et al (2019) [16] no.3**	Y	Y	Y	-	-	Y	-	Y	Y	Y	7
**Lu et al (2020) [17] no.1**	Y	-	Y	Y	-	Y	-	-	Y	Y	6
**Lu et al (2020) [17] no.2**	Y	-	Y	Y	-	Y	-	-	Y	Y	6
**Nikolaou et al (2020) [18] no.1**	Y	Y	Y	Y	-	-	-	-	Y	Y	6
**Nikolaou et al (2020) [18] no.2**	Y	Y	Y	Y	-	-	-	-	Y	Y	6
**Nikolaou et al (2020) [18] no.3**	Y	Y	Y	Y	-	-	-	-	Y	Y	6
**Sayour et al (2019) [19]**	Y	Y	-	Y	Y	Y	-	-	Y	Y	7
**Tanajak et al (2018) [20]**	Y	-	Y	Y	-	Y	Y	-	Y	Y	7
**Uthman et al (2019) [22] no.1**	Y	-	Y	Y	Y	Y	-	Y	Y	Y	8
**Uthman et al (2019) [22] no.2**	Y	-	Y	Y	Y	Y	-	Y	Y	Y	8

ESM Table 2. Modified CAMARADES study quality scores.

1: peer-reviewed publication; 2: control of temperature; 3: random allocation to treatment or control; 4: statement of confirmation of ischaemia; 5: blinded assessment of outcome; 6: measurement of cardiac function during ischaemia-reperfusion protocol; 7: animal model (aged, diabetic, or hypertensive); 8: sample size calculation; 9: compliance with animal welfare regulations; 10: statement of potential conflict of interests.

*The study by Jespersen et al. is a 'short communication', which might have compromised the reporting of study quality indicators.

Y=yes (reported); - =not reported

[13] Andreadou I, Efentakis P, Balafas E, et al. (2017) Empagliflozin Limits Myocardial Infarction in Vivo and Cell Death in Vitro: Role of STAT3, Mitochondria, and Redox Aspects. Front Physiol 8: 1077. 10.3389/fphys.2017.01077

[14] Baker HE, Kiel AM, Luebbe ST, et al. (2019) Inhibition of sodium-glucose cotransporter-2 preserves cardiac function during regional myocardial ischemia independent of alterations in myocardial substrate utilization. Basic Res Cardiol 114(3): 25. 10.1007/s00395-019-0733-2

[15] Lahnwong S, Palee S, Apaijai N, et al. (2020) Acute dapagliflozin administration exerts cardioprotective effects in rats with cardiac ischemia/reperfusion injury. Cardiovasc Diabetol 19(1): 91. 10.1186/s12933-020-01066-9

[16] Lim VG, Bell RM, Arjun S, Kolatsi-Joannou M, Long DA, Yellon DM (2019) SGLT2 Inhibitor, Canagliflozin, Attenuates Myocardial Infarction in the Diabetic and Nondiabetic Heart. JACC Basic Transl Sci 4(1): 15-26. 10.1016/j.jacbts.2018.10.002

[17] Lu Q, Liu J, Li X, et al. (2020) Empagliflozin attenuates ischemia and reperfusion injury through LKB1/AMPK signaling pathway. Mol Cell Endocrinol 501: 110642. 10.1016/j.mce.2019.110642

[18] Nikolaou PE, Efentakis P, Qourah FA, et al. (2020) Chronic Empaglifozin treatment reduces myocardial infarct size in non-diabetic mice through STAT-3 mediated protection on microvascular endothelial cells and reduction of oxidative stress. Antioxid Redox Signal. 10.1089/ars.2019.7923

[19] Sayour AA, Korkmaz-Icoz S, Loganathan S, et al. (2019) Acute canagliflozin treatment protects against in vivo myocardial ischemia-reperfusion injury in non-diabetic male rats and enhances endothelium-dependent vasorelaxation. J Transl Med 17(1): 127. 10.1186/s12967-019-1881-8

[20] Tanajak P, Sa-Nguanmoo P, Sivasinprasasn S, et al. (2018) Cardioprotection of dapagliflozin and vildagliptin in rats with cardiac ischemia-reperfusion injury. J Endocrinol 236(2): 69-84. 10.1530/JOE-17-0457

[21] Jespersen NR, Lassen TR, Hjortbak MV, et al. (2017) Sodium Glucose Transporter 2 (SGLT2) Inhibition does not Protect the Myocardium from Acute Ischemic Reperfusion Injury but Modulates Post-Ischemic Mitochondrial Function. Cardiovascular Pharmacology: Open Access 6(2). 10.4172/2329-6607.1000210

[22] Uthman L, Nederlof R, Eerbeek O, et al. (2019) Delayed ischaemic contracture onset by empagliflozin associates with NHE1 inhibition and is dependent on insulin in isolated mouse hearts. Cardiovasc Res 115(10): 1533-1545. 10.1093/cvr/cvz004

**Predictor variable**		**Subgroup SMD (95% CI)**	**Meta-regression (*p* for interaction)**	**Residual heterogeneity**
Anaesthetic regimen*			N/A	N/A
	With xylazine	-1.50 (-1.97, -1.03)		
	Without xylazine‡	N/A		
Use of oxygen*†			N/A	N/A
	Oxygen‡	N/A		
	No oxygen	-1.47 (-2.00, -0.93)		
Infarct size normalization method			*p*=0.006	T^2=0.33<br>I^2=46%
	Area at risk	-1.66 (-2.11, -1.22)		
	Total area	-0.53 (-1.50, 0.44)		

ESM Table 3. Subgroup analysis of selected predictor variables that were not prespecified.

*Only studies with *in vivo* myocardial ischaemia-reperfusion protocol are involved.

†If a study did not specify whether oxygen was used for the ventilation, the usage of room air was assumed (i.e. no oxygen).

‡Subgroup contains only 2 independent comparisons, therefore, total estimate, *p* for interaction and residual heterogeneity could not be calculated.

CI=confidence interval; N/A=not applicable; SMD=standardized mean difference

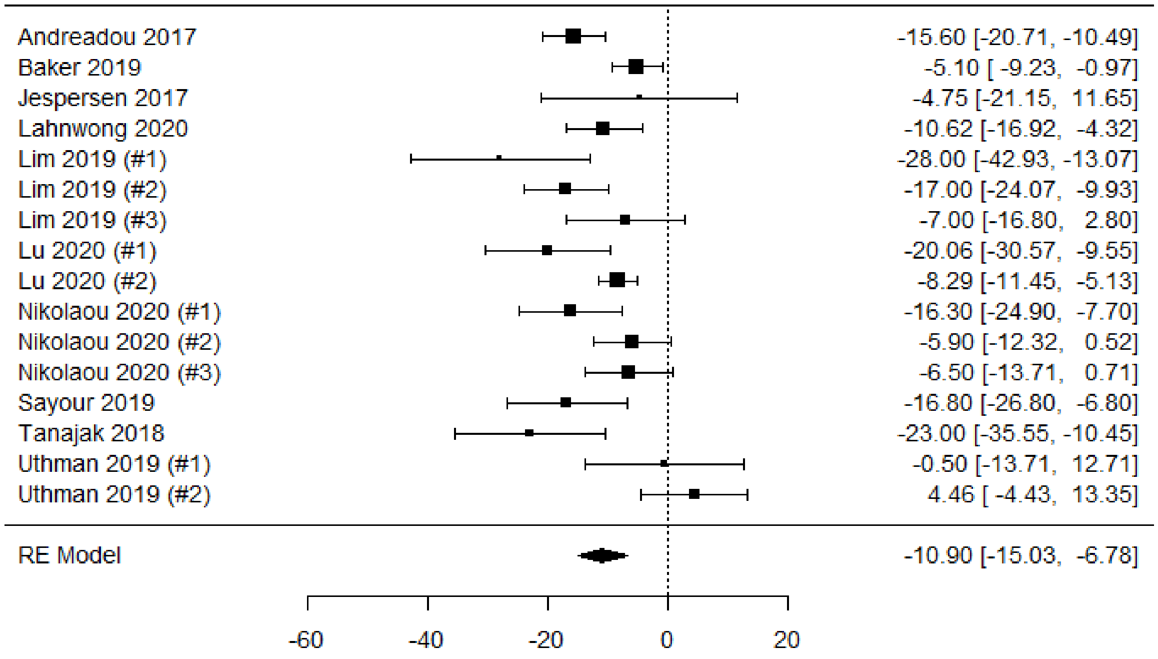

ESM Fig. 1. Sensitivity analysis using random-effects pooling method on weighted (unstandardized) mean differences (WMDs).

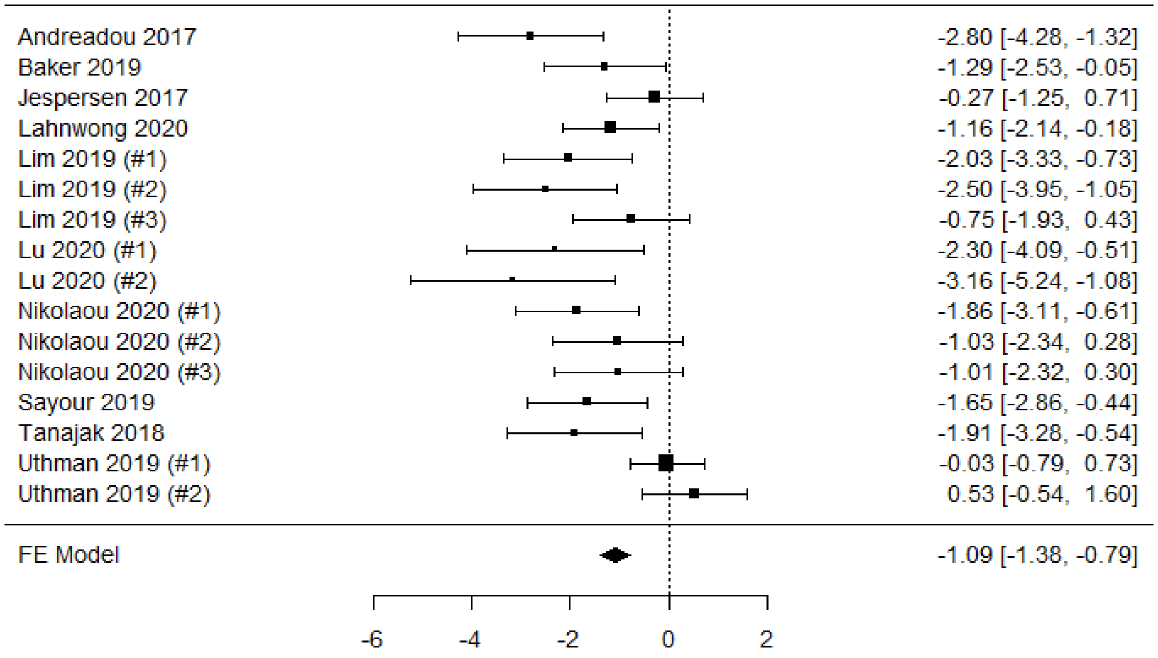

ESM Fig. 2. Sensitivity analysis using fixed-effects pooling method on standardized mean differences (SMDs).
